# Centromedian thalamic neuromodulation for the treatment of idiopathic generalized epilepsy

**DOI:** 10.3389/fnhum.2022.907716

**Published:** 2022-08-03

**Authors:** Andrew J. Zillgitt, M. Ayman Haykal, Ahmad Chehab, Michael D. Staudt

**Affiliations:** ^1^Department of Neurology, Beaumont Health Adult Comprehensive Epilepsy Center, Neuroscience Center, Royal Oak, MI, United States; ^2^Department of Neurosurgery, Beaumont Neuroscience Center, Royal Oak, MI, United States; ^3^Department of Neurosurgery, Oakland University William Beaumont School of Medicine, Rochester, MI, United States; ^4^Michigan Head and Spine Institute, Southfield, MI, United States

**Keywords:** anterior thalamic nucleus, centromedian nucleus, deep brain stimulation, epilepsy, idiopathic generalized epilepsy, responsive neurostimulation

## Abstract

Idiopathic generalized epilepsy (IGE) is a common type of epilepsy and despite an increase in the number of available anti-seizure medications, approximately 20–30% of people with IGE continue to experience seizures despite adequate medication trials. Unlike focal epilepsy, resective surgery is not a viable treatment option for IGE; however, neuromodulation may be an effective surgical treatment for people with IGE. Thalamic stimulation through deep brain stimulation (DBS) and responsive neurostimulation (RNS) have been explored for the treatment of generalized and focal epilepsies. Although the data regarding DBS and RNS in IGE is limited to case reports and case series, the results of the published studies have been promising. The current manuscript will review the published literature of DBS and RNS within the centromedian nucleus of the thalamus for the treatment of IGE, as well as highlight an illustrative case.

## Introduction

Epilepsy is defined as a disease of the brain characterized by an enduring predisposition to generate seizures. Epilepsy is defined by the presence of two or more unprovoked seizures separated by 24 h, one unprovoked seizure with a high probability for seizure recurrence, and/or the presence of a discrete electroclinical syndrome (Fisher et al., 2014). Epilepsy is the fourth most common neurologic condition with a prevalence in the United States of 1.2%, affecting around 3.4 million individuals (Zack and Kobau, 2017).

In a broad classification, generalized epilepsy is the second most common type of epilepsy after focal epilepsy. The term idiopathic generalized epilepsy (IGE) is used to describe a group of epilepsies with presumed genetic basis, manifesting as generalized-onset seizures, most commonly absence, myoclonic and/or generalized tonic-clonic seizures (GTCS). IGE syndromes share several clinical features including normal cognitive development, absence of gross neurological pathology, age dependency, and responsivity to certain anti-seizure medications (ASMs) (Andermann, 2006). Electrographically, they are characterized by the occurrence of generalized spike-and-wave discharges (GSWDs) or polyspike-and-wave discharges on an otherwise normal EEG background. Based on seizure types and age of onset, IGE can be further classified into syndromes, the most prevalent of which are childhood absence epilepsy (CAE), juvenile absence epilepsy (JAE), juvenile myoclonic epilepsy (JME) and IGE with GTCS alone (IGE-GTC).

Lennox-Gastaut syndrome (LGS) is an epileptic encephalopathy characterized by multiple seizure types including atypical absence, tonic-clonic, myoclonic, tonic and atonic seizures. Typically, EEG shows GSWDs and on a slow background. Due to its generalized clinical and EEG features, LGS was previously referred to as a “symptomatic generalized epilepsy,” a term that is no longer endorsed by the International League Against Epilepsy. LGS is often due to brain injury that almost always involves, but is not necessarily limited to, the cerebral cortex (such as hypoxic-ischemic encephalopathy, central nervous system infections, tuberous sclerosis complex, and metabolic disorders).

Despite an increase in the number of ASMs available to treat epilepsy seizures over the last 20–30 years, up to 20–30% of people with IGE are unable to control their seizures with medications alone (Kwan and Brodie, 2000; Kokkinos et al., 2020). Patients who continue to have seizures despite 2 adequately chosen and dosed ASMs are diagnosed with drug-resistant epilepsy (DRE) (Kwan and Brodie, 2000; Kwan et al., 2010; Chen et al., 2018). In IGE, there are several risk factors that may contribute to DRE, such as multiple seizure types (absence seizures, myoclonic seizures, GTCS), comorbid psychiatric disorders, generalized paroxysmal fast activity (GPFA) on EEG, catamenial seizures, high frequency of GTCS, and photosensitivity (Gelisse et al., 2001; Seneviratne et al., 2012; Cerulli Irelli et al., 2020; Pietrafusa et al., 2021; Kamitaki et al., 2022). Whereas surgical resection is often the optimal treatment for refractory focal epilepsy, patients with IGE are not candidates for resective surgery.

The first neuromodulation modality approved for the treatment of epilepsy was vagus nerve stimulation (VNS). VNS received FDA approval in 1997 for the treatment of focal-onset seizures (DeGiorgio et al., 2000), which was then extended to children age 4 and older in 2017 (Jain and Arya, 2021). In the United States, responsive neurostimulation (RNS) was approved by the FDA in 2013 for the treatment of focal epilepsy, while deep brain stimulation (DBS) of the anterior thalamic nucleus (ANT) was approved for the treatment of focal epilepsy in 2018 (2010 in Europe) (Ryvlin and Jehi, 2022).

Neuromodulation modalities have been utilized in the treatment of drug-resistant IGE. More recently, DBS and RNS targeting the centromedian nucleus (CM) of the thalamus have been explored as surgical therapies for people with drug-resistant IGE. Although VNS has been used in the treatment of drug-resistant IGE with some success (Kostov et al., 2007), this review will focus on the treatment of drug-resistant IGE through intracranial neuromodulation of the CM with DBS and RNS. An illustrative case is also provided to highlight the efficacy of CM-RNS in the treatment of IGE.

## Proposed generation of generalized epileptiform discharges and the centromedian nucleus of the thalamus

Although many thalamic nuclei may be involved in the generation of generalized epileptiform discharges and seizures, the CM and ANT appear to be activated during the initial spike-wave pattern in generalized epilepsy (Tyvaert et al., 2009).

GSWDs and generalized polyspike-and-wave discharges are the typical interictal signature of all IGE syndromes. Since the first description (Gibbs et al., 1935) of GSWDs in absence seizures, investigators have been intrigued by their distinctiveness from focal ictal patterns, particularly their abrupt onset and offset and synchronous bilateral distribution. This led to the speculation that GSWDs are generated by a midline subcortical pacemaker (Jasper and Kershman, 1941), possibly involving the diencephalon and mesencephalon (Penfield and Erickson, 1941). Studies in the 1940s demonstrated that patterns similar to GSWDs can be recorded over the cortex after stimulation of the thalamus in animals (Lewy and Gammon, 1940; Jasper and Drooglever-Fortuyn, 1947) and can be accompanied by clinical manifestations similar to human absence seizures (Hunter and Jasper, 1949). GSWDs were later recorded from thalami of patients with generalized epilepsy (Spiegel and Wycis, 1950; Williams, 1953).

Penfield and Jasper theorized that generalized-onset seizures originate in a *centrencephalic system* that includes the thalamus with the diencephalon and the brainstem (Penfield and Jasper, 1954), a concept dubbed as the *centrencephalic theory*. In the following decade, there were animal studies suggesting that GSWDs rather arise in the cortex (Marcus and Watson, 1966; Fischer-Williams et al., 1968) and can be triggered by electrical stimulation of the human frontal cortex (Bancaud et al., 1974), which sparked a long-standing debate between proponents of the *centrocephalic* and the *cortical focus* theories (Blumenfeld, 2005). Gloor subsequently introduced the theory of *generalized corticoreticular epilepsy* in which seizures are attributed to abnormal oscillations within corticoreticular networks (Gloor, 1968), and it eventually became widely accepted that thalamocortical network interactions are pivotal in generating typical generalized seizures (Blumenfeld, 2002; Avoli, 2012). Indeed, evidence from animal models suggested that neither cortex nor thalamus alone can generate GSWDs (Pellegrini et al., 1979; Avoli and Gloor, 1981; Danober et al., 1998; Blumenfeld, 2005). On the cellular level, it was suggested that reciprocal excitatory and inhibitory interaction between thalamocortical neurons and thalamic nucleus reticularis neurons give rise to the alternating excitatory (spike) and GABA_*A*_-mediated inhibitory (wave) activity during GSWDs (Blumenfeld, 2005).

More recently, evidence implicating thalamocortical networks emerged from magnetoencephalography studies (Amor et al., 2009; Stefan et al., 2009; Tenney et al., 2013) and from functional magnetic resonance imaging (fMRI) studies showing thalamic activation during GSWDs in patients with various IGE syndromes (Salek-Haddadi et al., 2003; Aghakhani et al., 2004; Moeller et al., 2008; Tyvaert et al., 2009). Importantly, one fMRI study examining thalamic nuclei activity during GSWDs in patients with CAE, JAE, and JME found that activation of the CM and parafasciular nuclei preceded that of the anterior nucleus, suggesting a predominant role of the CM-parafasciular complex in seizure generation or early propagation (Tyvaert et al., 2009). Studies of magnetic resonance spectroscopy suggested the presence of thalamic dysfunction in patients with JME and IGE with tonic-clonic seizures only, as evidenced by a reduction in thalamic N-acetyl aspartate/creatine ratio compared to controls in patients of mixed IGE syndromes (Bernasconi et al., 2003), a homogenous groups of patients with JME (Mory et al., 2003) and JAE (Kabay et al., 2010). Evidence of thalamocortical network dysfunction also came from a study using voxel-based morphometry and seed-based functional connectivity analysis in a mixed group of IGE patients (almost all with JME or IGE-GTC) (Kim et al., 2014).

GSWDs are also the hallmark of LGS, yet they typically occur at a lower frequency and are hence termed slow spike-and-wave discharges (SSWDs). Another electrographic signature of LGS is fast rhythmic waves or paroxysmal fast activity, often associated with tonic seizures (Blume, 2001; Verrotti et al., 2018). Although both patterns are primarily generated in the neocortex, the thalamus seems to be significantly involved (Blume, 2001). The neuronal substrates contributing to GSWDs in IGE and SSWDs in LGS are believed to be similar (Steriade, 2006). It has been shown that increased firing in the thalamocortical pathway may lead to a more sustained firing pattern of thalamic nucleus reticularis cells effecting conversion from GABA_*A*_- to GABA_*B*_-mediated inhibitory potentials of longer duration (Blumenfeld and McCormick, 2000). Accordingly, the greater cortical excitability in LGS compared to IGE may underlie the “slow” rate of SSWDs in LGS and its medical intractability (Blume, 2001).

The CM is considered a “diffuse-projecting” nucleus in the thalamus, with the lateral third of the CM exhibiting reciprocal connections with the premotor, motor, and primary somatosensory cortices (Ilyas et al., 2019). Tractography studies have also suggested the CM may be associated with the anterior insula and frontal operculum, with further projections to other thalamic nuclei, including the reticular nucleus (Ilyas et al., 2019). These diffuse projections may make it an ideal target for neuromodulation.

Defining the CM as a stereotactic target is primarily accomplished through indirect targeting based on the anterior commissure-posterior commissure line, as the thalamic nuclei are not visible on conventional imaging and intraoperative electrophysiology is not useful to guide placement. Accordingly, studies have attempted to define both optimal targeting techniques as well as success based on active contact location (Velasco M. et al., 2000; Velasco et al., 2007; Ilyas et al., 2022). Newer imaging modalities and improved MRI sequences may provide better delineation of the CM and allow for direct targeting in future studies (Warren et al., 2020; Middlebrooks et al., 2021).

## Centromedian nucleus-deep brain stimulation in generalized epilepsy

Whereas the treatment of focal seizures with ANT-DBS has received considerable attention and FDA approval (Fisher et al., 2010), the treatment of generalized seizures has yet to have an approved neuromodulation modality. There is a long history of CM stimulation for the treatment of intractable epilepsy, starting with reports by Velasco et al. describing the CM as a feasible and efficacious target (Velasco et al., 1987, 1995).

Three controlled trials assessing the efficacy of CM-DBS have been performed (Fisher et al., 1992; Velasco F. et al., 2000; Dalic et al., 2022). The first was a small study of 7 patients with drug-resistant focal epilepsy. There was a trend toward reduction of seizure frequency as a percentage of baseline, but this did not reach statistical significance (Fisher et al., 1992). In the study by Velasco et al., which included 15 patients with LGS, stimulation significantly decreased both the total number of seizures as well as the seizure subtypes, although no significant differences were identified when stimulation was turned off during the 3-month blinded follow-up (Velasco F. et al., 2000). A recent randomized controlled trial included 19 patients with LGS (Dalic et al., 2022). Half of the patient in the treatment group had ≥50% reduction in diary-recorded seizures after 3 months of stimulation, compared to 22% in the placebo group, but this difference was not statistically significant. A significant reduction in electrographic seizures on 24-h ambulatory EEGs was observed, a more objective measure. None of the aforementioned controlled trials included patients with IGE.

There have also been several small uncontrolled studies on the use of CM-DBS, most which focused on patients with LGS. Velasco et al. reported a significant difference in seizure reduction, and specifically >87% reduction in patients with appropriate electrode placement (Velasco et al., 2006). Son et al. reported a mean seizure reduction of 63.8% in a subset of four patients with LGS, compared to 69.2% in multilobar epilepsy, which was not significant (Son et al., 2016). Seizure reduction in a study reported by Kim et al. (2017) was 72% in a cohort of LGS and multifocal epilepsy. In a prospective, open label study on 20 patients with LGS or LGS-like syndrome, 90% were classified responders (Cukiert et al., 2020). Alcala et al. retrospectively reported a 60% in reduction in seizure frequency with simultaneous CM- and ANT-DBS compared with a 56% reduction in CM-DBS alone for the management of DRE in LGS or focal epilepsy (Alcala-Zermeno et al., 2021).

A recent systematic review of DBS targets in epilepsy reported a mean seizure reduction of 60.8, 73.4, and 67.8%, respectively with stimulation of the ANT, CMT, and hippocampus (Vetkas et al., 2022). The analysis included 8 studies of DBS-CMN with 90 patients in total. These authors suggested that the potentially most efficient DBS targets are the ANT for treatment of focal seizures, CM for generalized seizures, and hippocampus for temporal lobe seizures (Vetkas et al., 2022).

Reports on the use of CM-DBS for IGE are scarce; with the majority of reported cases being in patients with focal epilepsy, LGS or seizure types suggestive of LGS. There are only 6 reported cases of using CM-DBS in LGS. These are summarized in Table 1 (Cukiert et al., 2009; Valentin et al., 2013).

**TABLE 1:** Neuromodulation of the centromedian nucleus in the literature.

References	Age, sex	Seizure types	Pre-surgical testing	EEG findings	MRI findings	Pre-implantation seizure frequency	Current medications	Previous medication trials	Seizure reduction	Complications
**Deep brain stimulation**										
Cukiert et al. (2009)	36	Typical absence, tonic-clonic	MRI, EEG, SNAP-IV	Diffuse spike and wave 2.5 Hz	“MRI was normal in three patients and showed moderate diffuse atrophy in one”	Daily	Not reported	All patients were treated with at least high dose valproate, lamotrigine and phenobarbital in mono- or polytherapy before surgery	1 year: 70% reduction, 3×/week	No side-effects related to stimulation, although some transient contralateral paresthesias were present when voltage increases beyond 1.0 V
	24	Typical absence, tonic-clonic	MRI, EEG, SNAP-IV	Diffuse Spike and wave 3.0 Hz		Daily	Not reported		1 year: 85% reduction, 5×/week	
Valentin et al. (2013)	40, M	GTC, absence	EEG, MRI, neuropsychological examination, patient reported outcome questionnaire	Generalized epileptiform discharges, slow background	Normal	GTC—0.3 seizures/month Absence—1,000/day	Reduced dosage, levetiracetam, lamotrigine, carbamazepine, clobazam	Sodium valproate, levetiracetam, lamotrigine, carbamazepine, clobazam	72 months: Seizure-free since implantation (DBS off)	Device removed in one patient after 6 months due to infection Transitory agraphia during the first 4 days after implantation in one patient, completely resolved
	45, M	GTC, absence	EEG, MRI, neuropsychological examination, patient reported outcome questionnaire	Generalized epileptiform discharges, normal background	Normal	GTC—30/month Absence—30/day	Sodium valproate, levetiracetam, lamotrigine (reduced dose), clobazam	Sodium valproate, levetiracetam, lamotrigine, clobazam	66 months: 99% reduction in GTC, 100% reduction in absence	
	26, M	GTC, absence	EEG, MRI, neuropsychological examination, patient reported outcome questionnaire	Generalized epileptiform discharges, frontal slow	Normal	GTC—8/month Absence—12/day	Sodium valproate, levetiracetam, phenytoin,	Sodium valproate, levetiracetam, phenytoin, zonisamide	24 months: 25% reduction in GTC, 50% reduction in absence seizures	
	47, M	GTC, absence	EEG, MRI, neuropsychological examination, patient reported outcome, questionnaire	Generalized epileptiform discharges, normal background	Normal	GTC—2/month Absence—100/day	Zonisamide, levetiracetam, primidone	Zonisamide, levetiracetam, primidone	20 months: 87% reduction in GTC, 100% reduction in absence	
**Responsive neurostimulation**										
Kokkinos et al. (2020) and Sisterson et al. (2022)	19, F	Eyelid myoclonia with absences	EEG, MRI	Ictal EEG was characterized by 3–5-Hz generalized spikewave discharges, often incorporating polyspikes	Not reported	60/day	None at 33 months	Clobazam, ethosuximide, lamotrigine, levetiracetam, topiramate, zonisamide	84% reduction in seizures at 18 months, > 90% reduction at 33 months	None reported
Welch et al. (2021)	16, M	Childhood absence epilepsy	EEG, MRI	Scalp EEG: 3 Hz spike-and-slow-wave discharges, as well as interictal high-amplitude spike and polyspike-and-slow-wave discharges. SEEG: diffuse onset of 2.5–3.0 Hz spike-wave morphology; independent rare bursts of 2.5–3.0 Hz spike-wave discharges in right amygdala, never evolving to electrographic seizures	Lesion in the right amygdala suggestive of dysembryoplastic neuroepithelial tumor (lesion biopsied and ablated, although tissue not sufficient for diagnosis)	Daily typical absence seizures, with occasional progression to bilateral tonic-clonic seizure	Not reported	Ethosuximide, lamotrigine, topiramate, clobazam, valproate	6 months: 75% reduction	None reported
Sisterson et al. (2022)	20s, M	GTC, absence	EEG, MRI	Bursts of generalized spike/polyspike and wave discharges with right predominance	Not reported	Near-daily seizure frequency (mean 3/week), which would cluster Required intubation and ICU admission five times in 6 months prior to RNS implantation	Lacosamide, clobazam	Topiramate, levetiracetam, valproate, clonazepam, clobazam, zonisamide, oxcarbazepine/carbamazepine, lamotrigine	27 months: < 1 absence per month, < 1 GTC per year	None reported
	20s, F	Juvenile myoclonic epilepsy with GTC seizures and absences	EEG, MRI	Ictal EEG was characterized by generalized (maximal right frontal) 2.5–4.5 Hz spike/polyspike-and-wave discharges	Not reported	GTC 2–4 times/month, absence seizures few times per week	Brivaracetam	Lamotrigine, zonisamide, levetiracetam	25 months: GTC 1/month and less severe, no further absences, significant overall seizure frequency reduction of 75–89%	None reported
	30s, F	Myoclonic, absence, GTC	EEG, MRI	3.5–4.5 Hz generalized spike/polyspike and wave complexes	Not reported	Daily myoclonic seizures, weekly absences	Valproate, topiramate	Phenytoin, valproate, topiramate, lamotrigine, levetiracetam, clonazepam	24 months: <1 myoclonic per day, < 1 absence per week, < 1 GTC per year	None reported

Cukiert et al. (2009) reported on a series of 4 patients, 2 with IGE and 2 with LGS, who have previously undergone callosal section surgery prior to CM-DBS placement. The two patients with IGE reported a 70 and 85% reduction in seizure at 1 year post DBS-CMN. Additionally, the authors noted a clinically relevant increase in attention levels in all patients in an extended SNAP-IV questionnaire. In these patients, continuous stimulation was progressively increased by 0.2 V increments every 2 weeks until final parameters of 2 V, 130 Hz, and 300 μs were reached. Seizure frequency reduction was noted when simulation intensity reached 1.2 V, which progressively increased up to 2 V.

Valentin et al. (2013) reported the utility of CM-DBS in patients with IGE, LGS, and frontal lobe epilepsy. All 4 patients with IGE experienced a >50% reduction in seizure frequency during the blinded portion of the study. Interestingly, one patient remained seizure-free with the stimulator off at follow-up of 60 months following unblinding. Another patient was seizure-free for 1 year following implantation with the device turned off, then had 5 seizures in 1 month; following the initiation of stimulation, this patient remained seizure-free for 45 months. The blinded phase consisted of continuous stimulation of 60 Hz, 90 μs, and up to 5 V. Frequency of 130 Hz was initially used, although was adjusted to 60 Hz following unclear clinical efficacy, and to be consistent with previous CM-DBS studies (Fisher et al., 1992).

## Responsive neurostimulation in generalized epilepsy

Unlike DBS, RNS is a technology that operates in a closed-loop circuit, via detection of epileptic discharges that are defined based on the patient’s unique electrographic seizure characteristics. When these preset discharges are recognized and detected by the device, responsive therapy is delivered via electrical current. Therefore, RNS provides individualized therapy that can be modified and adjusted based on the patient’s electrocorticography (ECoG) data and seizures. The publication of the RNS pivotal trial in 2011 (Morrell, 2011) resulted in FDA approved for its use in patients ≥18 years with ≤2 seizure foci. There has since been substantial interest in targeting the CM with RNS for the treatment of drug-resistant IGE; however, the data regarding the use of RNS in IGE are limited. There are only 2 case reports and a single case series illustrating the potential efficacy of this therapy (Table 1; Kokkinos et al., 2020; Welch et al., 2021; Sisterson et al., 2022).

In the first reported case, a 19-year-old female with eyelid myoclonia with absences underwent bilateral CM-RNS implantation (Kokkinos et al., 2020). Following implantation, her seizures decreased from a mean of 60 seizures per day to less than 10 seizures per day at 18-month follow-up. Interestingly, after RNS implantation within the bilateral CM, she retained awareness during her absence seizures. This patient was reported on in a subsequent publication (Sisterson et al., 2022), with greater than 90% seizure frequency reduction maintained at 33-month follow-up. Stimulation of 1–2 mA, 125 Hz, 160 μs for 5,000 ms was employed. Three additional patients in this series with IGE underwent bilateral CM-RNS implantation, with similar significant reduction in seizure frequency that was sustained between 24–27 months of follow-up (Sisterson et al., 2022). Stimulation amplitude varied between 0.2 and 2.0 mA between patients, with amplitude increasing over successive epochs; however, patients received differing amounts of overall daily stimulation and total charge duration. All stimulation paradigms were delivered in bursts lasting 5,000 ms.

In another case, a 16-year-old male childhood absence epilepsy underwent bilateral CM-RNS (Welch et al., 2021). After implantation and turning on stimulation, seizure frequency was improved, although with continued multiple weekly absence seizures. At 6-month follow-up, there was resolution of his daily absence seizures. In addition, the frequency of his GTCS decreased from 3–4 per month to 1 per month. The stimulation parameters at 6 month were 1.5 mA, 125 Hz, 160 μS for 5,000 ms.

In addition to focal epilepsy and IGE, other studies have evaluated different indications for the use of CM-RNS as well. Burdette et al. (2020, 2021) have demonstrated the utility of thalamic stimulation in regional neocortical epilepsy with RNS. In one study of neocortical epilepsies, one depth lead was implanted into the CMT and the other lead into the most active neocortical region, with 67% mean reduction in all seizures and 80% reduction in disabling seizures over 17 months (Burdette et al., 2020). In another unique case series of posterior quadrant epilepsies, one lead was placed in the pulvinar nucleus with a second lead over the ipsilateral posterior quadrant, with all patients having ≥50% seizure reduction and 2 of 3 patients having ≥90% seizure reduction at last follow-up (Burdette et al., 2021). Bilateral CM-RNS has also been demonstrated to significantly improve seizure frequency in pediatric patients with LGS and autism spectrum disorder (Kwon et al., 2020).

## Illustrative case

The patient is a 41-year-old right-handed female with a history of drug resistant IGE manifesting as typical absence seizures, myoclonic seizures, GTCS. She had a family history of epilepsy (father with focal epilepsy). There were no other epilepsy risk factors. Her seizures began at the age of 4 years old. Over time, she continued to experience frequent GTCS that were often followed by non-convulsive status epilepticus (NCSE, absence status epilepticus). At the time of her initial evaluation in our comprehensive epilepsy center, she would have at least 1 GTCS per year but often would have 1 GTCS every 2–3 months. From 2018 to 2020, she had 11 GTCS that were consistently followed by 1–2 days of NCSE, requiring hospital admissions. Throughout her epilepsy course, she failed medication trials of levetiracetam, topiramate, perampanel, and ethosuximide.

**FIGURE 1:**
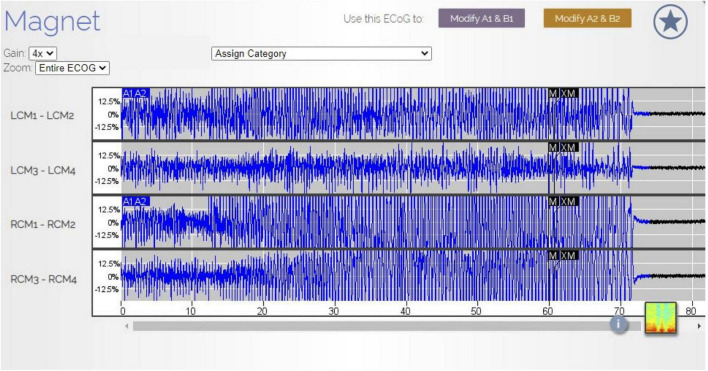
RNS electrothalamogram (ETG) of GTCS. In August 2021, the patient experienced a GTCS. Her family used the RNS magnet (black labels M and XM in the ETS) to store this ETS data in the patient data management system (PDMS). The ETG represents 1 long episode (LE) lasting 90 s. The A1A2 blue labels are the RNS detection of this seizure. The seizure is characterized by rhythmic, beta range, spike-wave discharges (Gain 4×). Note, the patient was not undergoing scalp EEG at the time of this seizure and no direct comparisons between scalp EEG findings and RNS ETS were available.

In light of her DRE, she was evaluated for epilepsy surgery. A brain MRI was unremarkable for discrete epileptic lesions. Inpatient video-EEG monitoring revealed generalized, frontally predominant, 3–4 Hz, spike- and polyspike-and-wave discharges as well as bursts of GPFA (Figure 1). Her case was reviewed at our patient management conference. Considering her history, semiology, and diagnostic studies, resective surgery was not a reasonable treatment option; however, neuromodulation was thought to be a reasonable treatment consideration. Our experience with VNS in IGE was underwhelming, while our experience with RNS and DBS (albeit in focal epilepsy) was quite positive. Overall, our group favors the ability of RNS to record ECoG and adjust detection and treatment parameters based on these data. In light of new case reports illustrating the potential safety and efficacy of RNS in people with IGE, neuromodulation was offered and she elected to pursue RNS implantation. She then underwent RNS implantation in February 2021 with depth electrodes placed into the bilateral CM. At the time of RNS implantation, her medication regimen consisted of lamotrigine, brivaracetam, cannabidiol, and clonazepam polytherapy.

**FIGURE 2:**
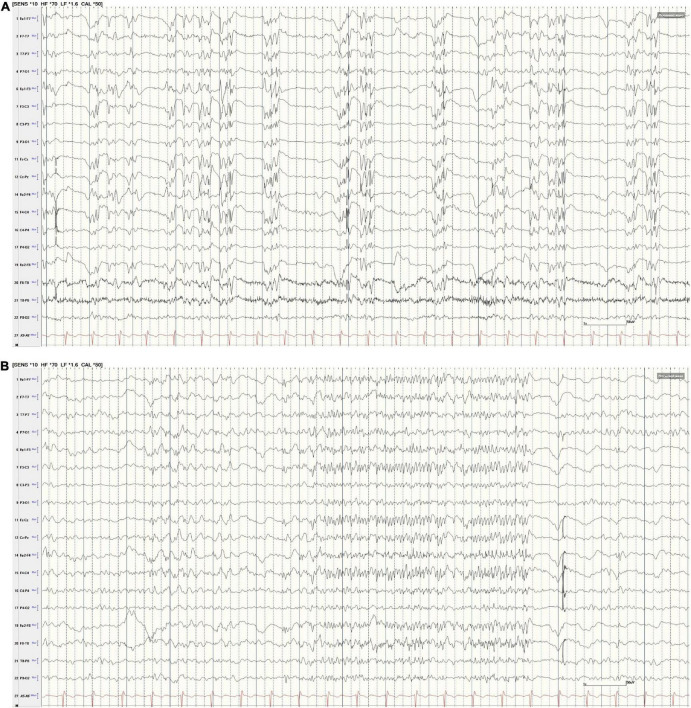
**(A,B)** Scalp EEG following GTCS. **(A)** AP Bipolar montage (15 s; HFF 70 Hz, LFF 1.5 Hz, Sen 10 uV). The tracing reveals nearly continuous generalized polyspike-and-wave discharges. At times, these discharges were intermixed with generalized paroxysmal fast activity (GPFA, **B**). Note, the ETG captured by RNS and illustrated in Figure 3 do not correspond to these specific scalp EEG examples in **(A,B)**.

**FIGURE 3:**
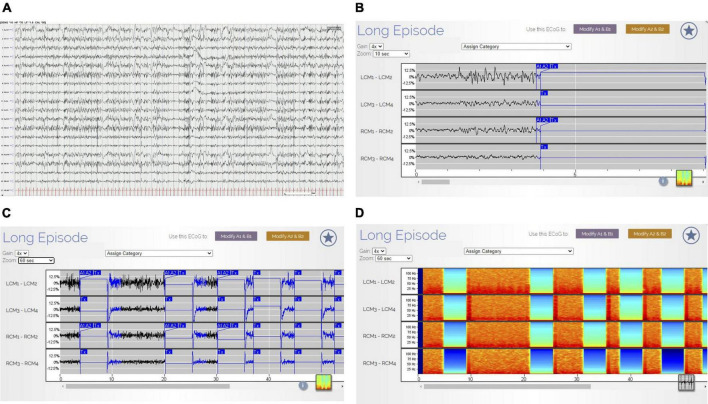
**(A–D)** Non-convulsive status epilepticus (NCSE) on scalp EEG and RNS Findings. The tracing in **(A)** (AP Bipolar montage, 60 s; HFF 70 Hz, LFF 1.6 Hz, Sen 10 uV) illustrates continuous generalized polyspike-and-wave discharges. Clinically, the patient exhibited impaired cognition and responsiveness. There were no motor features during these EEG changes. The ETG in RNS **(B–D)** corresponds directly to the EEG 60 s epoch in **(A)**. B reveals the first 10 s of ETG data (gain 4×) corresponding to the first 10 s of scalp EEG data displayed in **(A)**. There are continuous, spike-wave discharges within the left and right CMN contacts. **(C)** (Gain 4×) reveals the 60 s of ETG data corresponding to the 60 s of scalp EEG data. The blue A1/A2 boxes denote RNS detection followed by a delivered therapy (Tx). During therapy, there is interruption in the ETS background. In **(D)** (gain 4×), the density spectral array demonstrates bursts of high-power activity that corresponds to the ictal activity (rhythmic, beta range spike-waves) in the ETG tracing **(C)**. The low power (blue/green) in the density spectral array corresponds to delivered therapies (identified in **(C)** by the Tx blue box).

Initially, following RNS implantation, she continued to experience frequent GTCS with approximately 1 GTCS every 1–3 months. Six months after RNS implantation, she experienced a cluster of GTCS that was followed by absence status epilepticus (Figures 1–3). During the subsequent hospital admission, she was treated with intravenous lacosamide (400 mg loading dose, followed by 200 mg twice daily). An adjunctive trial of valproate was initiated (2,000 mg loading dose and 1,000 mg twice daily). Her RNS therapy settings were also changed (Table 2). No changes were made to her detection parameters. Following treatment with lacosamide and valproate and changes to her RNS therapy settings, her seizures resolved; however, with time, treatment with lacosamide and valproate was poorly tolerated. These medications were tapered and discontinued. No changes were made to her RNS therapy settings during these medication changes. She has remained seizure-free for 10 months with her current treatment regimen. In addition, on review of her RNS data, she has experienced a marked reduction in the number of long episodes (LEs) per month and therapies per day. Prior to the RNS changes, she would have 98 LEs per month with 80 therapies per day (total of 154 days of treatment). After the RNS changes, her LEs decreased to 0.2 per month and her therapies per day decreased to 15 per day (total of 287 days of treatment).

**TABLE 2:** RNS Detection and therapy settings.

Detection settings	Minimum frequency (Hz)	Maximum frequency (Hz)	Minimum amplitude (%)	Minimum duration (s)	
Pattern A1 (Bandpass)	2 (spiking)	15.63 (sinusoid)	6.26	0.512	
Pattern A2 (Bandpass)	2 (spiking)	15.63 (sinusoid)	5.47	0.512	
**Therapy settings**	**Current/prior setting (mA)**	**Frequency/prior setting (Hz)**	**Pulse width/prior setting (μs)**	**Burst duration/prior setting (ms)**	**Charge density/prior setting (μC/cm^2^)**
Burst 1	1.5/1	25/125	160/160	5,000/5,000	0.8/0.5
Burst 2	Off	Off	Off	Off	Off

Therapy settings were tested with a stepwise approach. Initially, the current and charge density were increased. She tolerated these initial adjustments. There were then reductions to her frequency settings. There were no after discharges during stimulation. There were no adverse reactions, e.g., paresthesia. The changes to her therapy settings were based on our clinical experience with 4 other patients with IGE and RNS.

## Limitations of reported literature

To date, the published literature regarding neuromodulation in IGE is primarily limited to case reports and case series. As such, there are many questions related to patient selection, safety, neuromodulation settings, and outcomes. Although the failure of two appropriately chosen medications at appropriate doses denotes drug resistant epilepsy, should patients with IGE have a trial of valproic acid before proceeding to surgery? Are certain electroclinical syndromes more responsive to neuromodulation, e.g., epilepsy with generalized tonic-clonic seizures alone? What are the optimal DBS and RNS settings for IGE? For RNS, the baseline simulation settings in previously published reports have largely adhered to those established for ANT-DBS (Fisher et al., 2010); however, the heterogeneity in individual settings and treatment outcomes warrants further exploration.

## Conclusion and future directions

Drug resistance is common and may be present in up to 20–30% of IGE, and unfortunately there is no FDA-approved treatment for IGE. Although historically surgical options for IGE have been limited, there is increasing evidence that neuromodulation may be an effective treatment option for these patients. Case series utilizing DBS for IGE have been reported and more recently, case reports illustrating the utility of RNS in treating IGE have been published. Bilateral CM-RNS offers an exciting new treatment paradigm in which detection of epileptiform activity in the brain is possible and therapy may be tailored specifically for each patient. The initial promising results from bilateral CM-RNS for the treatment of IGE has led to the initiation of a prospective single blind, multi-center, randomized study (NAUTILUS).^1^ As case reports and series of CM-RNS in IGE continue to grow and the results of randomized controlled clinical trials become available, RNS may emerge as a viable treatment option for people with drug-resistant IGE.

## Author contributions

AZ and MS contributed to conception and design of the review. AZ wrote the case illustration. MS provided supervision and project administration. All authors performed the literature review, contributed to drafting of the manuscript, and read and approved the submitted version.

## Footnotes

1
https://clinicaltrials.gov/ct2/show/NCT05147571
